# A Combination of Soy Isoflavone and L-Carnitine Improves Running Endurance in Mice

**DOI:** 10.3390/nu15173678

**Published:** 2023-08-22

**Authors:** Jaewon Lee, Yoonjoe Joh, Cheoljun Choi, Kyungmin Kim, Yun-Hee Lee

**Affiliations:** College of Pharmacy and Research Institute of Pharmaceutical Sciences, Seoul National University, Seoul 08826, Republic of Korea; ljo2291@snu.ac.kr (J.L.); libertines321@snu.ac.kr (Y.J.); hgg121@snu.ac.kr (C.C.); crystall26@snu.ac.kr (K.K.)

**Keywords:** soy isoflavone, L-carnitine, running endurance, AMPK

## Abstract

The present study aimed to investigate the effect of APIC, a mixture containing soy isoflavone and L-carnitine on running endurance. Male C57BL/6 mice were orally administered APIC for 8 weeks. The APIC group exhibited a significant increase in treadmill running time until exhaustion compared to the control group. The respiratory exchange ratio in the APIC group was lower, indicating an enhancement in fatty acid oxidative metabolism. Furthermore, APIC supplementation increased the proportion of oxidative myofibers. Biochemical parameters associated with endurance capacity were also affected by APIC, as evidenced by increased muscle ATP levels and decreased levels of muscle triglycerides and blood lactate. qPCR and immunoblot analysis of C2C12 myotubes and gastrocnemius muscles indicated that APIC treatment stimulated AMPK signaling, mitochondrial biogenesis, and fatty acid metabolism. Additionally, treatment with APIC led to an increased oxygen consumption rate in C2C12 myotubes. Collectively, these findings suggest that APIC supplementation enhances mitochondrial biogenesis, promotes a switch from glycolytic to oxidative fiber types, and improves fatty acid metabolism through the activation of the AMPK signaling pathway in murine skeletal muscle. Ultimately, these effects contribute to the enhancement of running endurance.

## 1. Introduction

A sedentary lifestyle is a major risk factor for health in modern times. Regular exercise enhances longevity by boosting overall muscular strength and endurance while offering defense against various ailments [1,2].

Skeletal muscle is composed of heterogenous myofibers that contain slow-twitch (type I) and fast-twitch (type IIa, IIx, and IIb) fibers. Type I myofibers express myosin heavy chain (MyHC) isoform I, exhibit a significant abundance of mitochondria and remarkable oxidative capacity, and are resistant to fatigue [3]. On the other hand, type IIb myofibers express MyHC IIb, have low mitochondrial content and high glycolytic enzyme activity, and are prone to fatigue. MyHC isoform IIa and MyHC IIx display intermediate characteristics between MyHC I and MyHC IIb. MyHC IIa possesses a higher oxidative capacity compared to MyHC IIx [3,4]. Skeletal muscle is a highly plastic organ that undergoes myofiber-type transformations in response to various external stimuli, including exercise [5]. For example, endurance exercise can induce a transition from fast-twitch to slow-twitch fiber types [6,7]. Consequently, a viable strategy for improving exercise endurance involves targeting the transition from fast-twitch to slow-twitch fiber types [8].

Enhancing fatty acid oxidation is a crucial mechanism for improving exercise endurance. By increasing fatty acid oxidation, the body shifts its primary fuel source from glucose to fatty acids. This shift reduces the dependence on glucose for energy production [9]. Carnitine palmitoyltransferase 1 (CPT1) is a key enzyme involved in the process of fatty acid oxidation. Working in conjunction with L-carnitine, CPT1 facilitates the transport of long-chain fatty acids into the mitochondria. Once inside the mitochondria, these fatty acids undergo oxidation to produce energy [10]. Several studies have demonstrated the beneficial effect of L-carnitine on improving endurance exercise performance by increasing fat oxidation [11,12], although its efficacy remains controversial [13,14].

Exercise training activates 5′-adenosine monophosphate-activated protein kinase (AMPK) in skeletal muscle [15,16]. AMPK plays a crucial role in regulating energy production by inhibiting anabolism and promoting catabolism. One of its key actions is the promotion of fatty acid oxidation by phosphorylating acetyl-CoA carboxylase (ACC) [17]. ACC is responsible for the synthesis of malonyl-CoA, which inhibits CPT1 [18]. Additionally, AMPK is involved in the stimulation of mitochondrial biogenesis [19], leading to an increase in the number and metabolic activity of mitochondria within skeletal muscle. Notably, studies have reported that the AMPK agonist AICAR can enhance running endurance in sedentary mice [16].

Isoflavones, derived from soybeans, belong to the flavonoid family. They are recognized as phytoestrogen due to their molecular structure and resemblance to the female hormone estrogen [20]. Isoflavones are suggested to offer beneficial effects in preventing various diseases, including cancer, cardiovascular disease, osteoporosis, and menopausal syndrome [20]. Notably, our previous study demonstrated that soybean embryo extract, rich in isoflavones, exhibits an anti-obesity effect by promoting the browning of adipose tissue [21]. Furthermore, several studies have reported the ability of isoflavone to activate AMPK, a key cellular energy sensor [22,23].

Previous studies have reported a potential synergistic effect of isoflavone and L-carnitine in improving fatty acid metabolism. Specifically, treatment of human hepatoma HepG2 cells with genistein or daidzein resulted in elevated gene expression levels and enzyme activity of CPT1. Notably, the combined treatment of genistein or daidzein with L-carnitine further enhanced the activity of CPT1 [24]. In vivo experiments using mice fed a high-fat diet (HFD) and administered a mixture of soy isoflavone and L-carnitine showed significant reductions in triglyceride and total lipid levels in the liver. Furthermore, both the soy isoflavone and L-carnitine groups exhibited increased protein expression of CPT1 in the liver. These findings suggest that the supplementation of either soy isoflavone or L-carnitine can effectively modulate liver lipid metabolism and enhance CPT1 expression in the context of a high-fat diet [25].

In this study, we aimed to investigate the effect of a combined treatment of soy isoflavone and L-carnitine on running endurance, as well as to elucidate the underlying mechanism involved.

## 2. Materials and Methods

### 2.1. Materials

A combination of soybean embryo extract and L-carnitine in a 4:3 ratio, known as AmorePacific Isoflavone & Carnitine (APIC), was provided by AMOREPACIFIC Corp. (Seoul, Republic of Korea). APIC contains soy isoflavone, which constitutes 5.1 ± 0.5% of total weight. Isoflavones are composed of 10% genistin (genistein), 40% glycitin (glycitein), and 50% daidzin (daidzein) [21]. L-carnitine content in APIC is 42.4 ± 2.4%.

### 2.2. Animals

All the animal study protocols were reviewed and approved by the Institutional Animal Care and Use Committees of Seoul National University (SNU-220209-2). Six-week-old male C57BL/6J mice were purchased from Joongah Bio (Suwon, Gyeonggi-do, Republic of Korea), housed under a 12 h light/12 h dark cycle condition at 22 ± 1 °C, with free access to food and water. The mice were divided into groups and orally administered a high dose (200 mg/kg, 200 A) or a low dose (100 mg/kg, 100 A) of APIC dissolved in distilled water once a day for eight weeks. Body weight was measured weekly throughout the experiment. 

### 2.3. Exhaustive Treadmill Test

The exhaustive treadmill test was performed on a treadmill (Hwayon Biotech, Shenzhen, China) with a 5° incline. The treadmill initially ran at 12 m/min for 10 min. Subsequently, the speed was increased by 2 m/min every 5 min up to 24 m/min and then maintained constant until exhaustion. Exhaustion was defined as the inability to run despite the electrical stimulation (1.0 mA). The running time and distance were recorded to assess endurance capacity. The test was conducted during the day, starting at 3 PM.

### 2.4. Indirect Calorimetry and Body Composition

The respiratory exchange ratio (VO_2_/VCO_2_) and food intake were measured using the PhenoMaster (TSE system, Bad Homburg, Germany) under the conditions of 24 °C and 12 h light/12 h dark cycle with free access to food and water [26]. The body composition of the mice was assessed and measured using nuclear magnetic resonance (NMR) scanning EchoMRI-700 (EchoMRI, Houston, TX, USA) [26].

### 2.5. Immunofluorescence Staining

After treadmill running, the mice were sacrificed, and their gastrocnemius muscles were embedded in the OCT compound (Leica, Wetzlar, Germany) and then frozen in liquid nitrogen-cooled isopentane. Using a cryostat (Leica), 10 μm thick cryosections were prepared. For staining, the cryosections were blocked with 3% bovine serum albumin (BSA) in DPBS for 1 h at room temperature and then incubated overnight at 4 °C with a cocktail of primary antibodies, including MyHC I (BA-D5, DSHB, Iowa City, IA, USA), MyHC IIa (SC-71, DSHB), MyHC IIb (BF-F3, DSHB), and Laminin (ab11575, Abcam, Cambridge, UK). Following the wash step, the cryosections were incubated with a cocktail of secondary antibodies (Alexa Fluor 647-conjugated goat anti-mouse IgG2b (A21242), Alexa Fluor 568-conjugated goat anti-mouse IgG1 (A21124), Alexa Fluor 488-conjugated goat anti-mouse IgM (A21042), and Alexa Fluor 405-conjugated goat anti-rabbit IgG (A31556), Thermo Fisher Scientific, Waltham, MA, USA) for 1 h at room temperature. Images of the cryosections were acquired using a Leica Thunder imager 3D Assay (Leica Application Suite X (LAS X) 3.6.0; Leica) and analyzed using Myosoft (version 15) [27], an automated muscle histology analysis software.

### 2.6. Cell Culture

C2C12 myoblast cells (ATCC, Manassas, VA, USA) were cultured in Dulbecco’s Modified Eagle’s Medium (DMEM, Welgene, Gyeongsan, Republic of Korea) containing 10% fetal bovine serum (FBS, Gibco, Grand Island, NY, USA). At a confluency of 90–100%, the medium was replaced with a differentiation medium consisting of DMEM with 2% horse serum (HS, Gibco) and the cells were cultured for 4 days. After the 4-day culture with 2% HS, the cells were treated with vehicle (DPBS) or APIC (80 µg/mL, 80 A; 160 µg/mL, 160 A) for 24 h.

### 2.7. Immunoblot Analysis

Immunoblot analysis was conducted as previously mentioned [28]. Cells were lysed with RIPA Buffer (Thermo Fisher Scientific) containing protease inhibitor (Sigma-Aldrich, St. Louis, MO, USA) and phosphatase inhibitor (Roche, Basel, Switzerland). Gastrocnemius muscle samples were homogenized using PRO-PREP Protein Extraction Solution (iNtRON Biotechnology, Seongnam, Republic of Korea) containing protease inhibitor (Sigma-Aldrich, St. Louis, MO, USA) and phosphatase inhibitor (Roche, Basel, Switzerland). The Pierce BCA Protein Assay Kit (Thermo Fisher Scientific) was used to determine protein concentration. Proteins were separated on a 12% SDS–PAGE gel and were transferred to an Immun-Blot PVDF membrane (Bio-Rad, Hercules, CA, USA). The membrane was then incubated for 1 h at room temperature in a blocking buffer (5% BSA or 5% powdered skim milk in TBST) and subsequently incubated with primary antibodies at 4 °C overnight. The primary antibodies used are as follows: pAMPK (2531S, Cell Signaling Technology, Beverly, MA, USA), AMPK (2532S, Cell Signaling Technology), pACC (3661S, Cell Signaling Technology), ACC (3662S, Cell Signaling Technology), PGC-1α (AB3242, Millipore, Danvers, MA, USA), GAPDH (sc-32233, Santa Cruz Biotechnology, Dallas, TX, USA), Total oxphos (ab110413, Abcam), Citrate synthase (14309S, Cell Signaling Technology), PPARβ/δ (sc-74517, Santa Cruz Biotechnology). After the primary antibody incubation, the membrane was washed and incubated for 1 h at room temperature with horseradish peroxidase-conjugated secondary anti-mouse or anti-rabbit (Thermo Fisher). Blots were visualized by the Fusion Solo chemiluminescence imaging system (Vilber Lourmat, France) and analyzed with EvolutionCapt software (version 17.03). ImageJ (National Institutes of Health, Bethesda, MD, USA) was utilized for quantifying the band intensity.

### 2.8. Quantitative PCR

Quantitative PCR (qPCR) was performed as mentioned previously [29]. Total RNA was extracted from gastrocnemius muscles using TRIzol reagent (Invitrogen, Waltham, MA, USA) and cDNA was synthesized using High-Capacity cDNA Reverse Transcription kit (Applied Biosystems, Waltham, MA, USA). qPCR reaction was conducted with iQ SYBR Green Supermix (Bio-Rad) in CFX Connect Real Time PCR Detection system (Bio-Rad). Peptidylprolyl isomerase A (*Ppia*) was used as an internal reference. Primers used for qPCR are listed in Table 1.

### 2.9. Triglyceride Assay

Intramuscular triglyceride was measured using Triglyceride Colorimetric Assay Kit (Cayman Chemical, Ann Arbor, MI, USA) following the manufacturer’s instructions. Briefly, 50 mg of gastrocnemius was homogenized in 250 μL of NP40 Reagent with protease inhibitor (Sigma-Aldrich). The supernatant was transferred to a fresh tube after centrifuging tissue homogenates at 10,000× *g* for 10 min at 4 °C. Samples were diluted to the ratio of 1:5, and then incubated with reaction mixture for 60 min at room temperature. The absorbance was read at 545 nm using MultiSkan GO spectrophotometry (Thermo Fisher Scientific).

### 2.10. ATP Assay

Intramuscular ATP was measured using ATP Assay Kit (Abcam) according to the manufacturer’s instructions. Briefly, 20 mg of gastrocnemius was homogenized in 200 μL of ice-cold 2 N PCA and incubated on ice for 30 min. The supernatant was transferred to a fresh tube after centrifuging tissue homogenates at 13,000× *g* for 2 min at 4 °C. Sample volumes were diluted to 500 μL with the ATP assay buffer. To precipitate excess PCA in the sample, 50 μL of ice-cold 2 M KOH was added, and then the sample was adjusted to pH 6.5~8 with 2 M KOH and 2 N PCA. Samples with adjusted pH were centrifuged at 13,000× *g* for 15 min at 4 °C and supernatants were collected. Next, 4 μL/well of samples were loaded onto 96-well plates and then ATP assay buffer was added to adjust the volume to 50 μL/well. For the reaction, 50 μL of the reaction mixture was added to sample wells and incubated at room temperature for 30 min shielded from any exposure to light. For the background controls, 50 μL of background reaction mix was added instead of the reaction mix. The absorbance was read at 570 nm using MultiSkan GO spectrophotometry (Thermo Fisher Scientific).

### 2.11. Measurement of Plasma Lactate Level

Plasma lactate level was measured using StatStrip Xpress Lactate Meter (Nova Biomedical, Waltham, MA, USA). Immediately after the final treadmill running exercise, a single drop of blood was obtained from the tail tip and applied onto a disposable strip for lactate measurement.

### 2.12. Measurement of Oxygen Consumption Rates

Oxygen consumption rates (OCRs) were measured as previously mentioned [28] by the Seahorse XF Analyzers (Agilent, Santa Clara, CA, USA). OCRs were normalized with protein concentrations. Basal and maximal respiration was calculated by subtraction of non-mitochondrial respiration. Proton leak was calculated by subtracting non-mitochondrial respiration from the oligomycin A-induced OCR. ATP production-related respiration was calculated by subtracting the oligomycin A-induced OCR from the basal respiration.

### 2.13. Statistical Analysis

Data are presented as mean ± standard error of the mean (SEM). Statistical significance between the two groups was determined by unpaired *t*-test. A *p*-value less than 0.05 was considered to be statistically significant. Statistical analyses were calculated by GraphPad Prism 7 (ver. 7.0, GraphPad Software, Boston, MA, USA).

## 3. Results

### 3.1. APIC Supplementation Enhances Running Endurance

**Figure 1:**
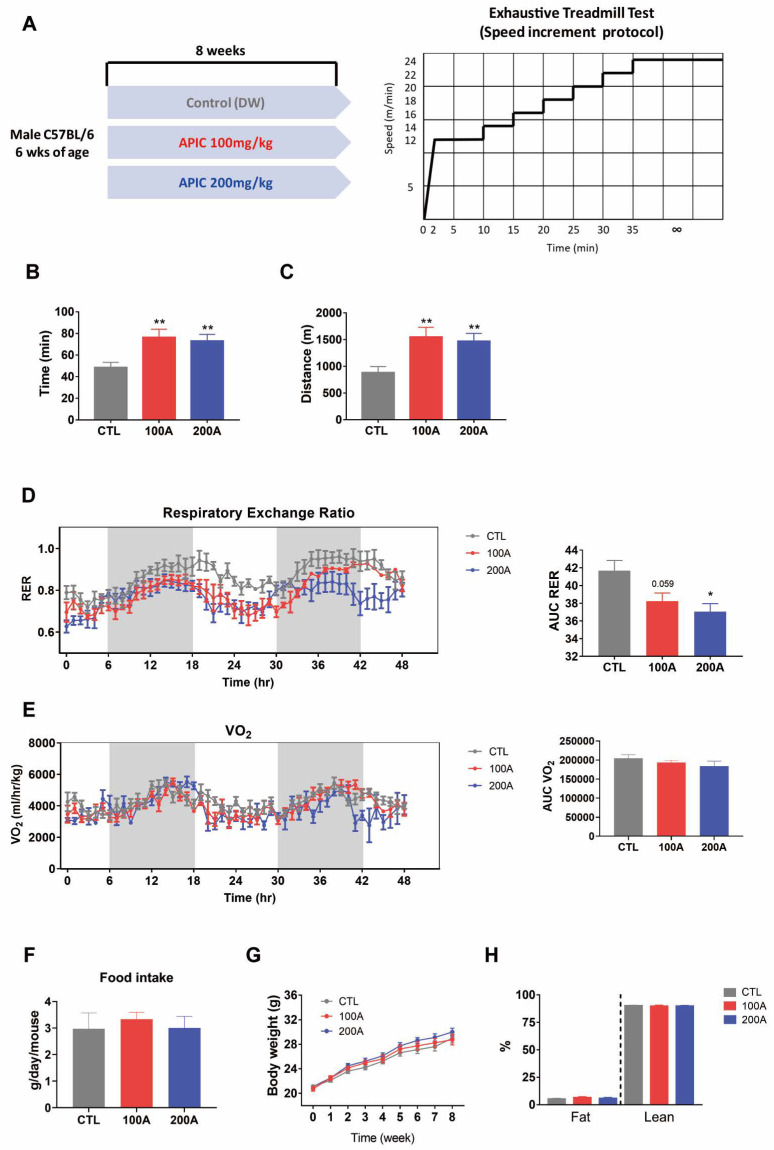
Effects of APIC supplementation on running endurance and lipid utilization. (**A**) A schematic diagram of the experimental design and the incremental protocol used for the exhaustive treadmill test. (**B**,**C**) Running time and distance until exhaustion were measured under an exhaustive treadmill test. (**D**,**E**) Indirect calorimetry analysis (*n* = 4). RER: respiratory exchange ratio; VO_2_: rate of oxygen consumption. (**F**) Daily food intake (*n* = 4). (**G**) Body weight monitoring (*n* = 8). (**H**) Body composition (*n* = 8). Values are mean ± SEM. * *p* < 0.05 and ** *p* < 0.01. CTL, vehicle; 100 A, APIC 100 mg/kg/day; 200 A, APIC 200 mg/kg/day.

The mice were orally administered a combination of soy isoflavone and L-carnitine, specifically APIC, at doses of 100 mg/kg/day and 200 mg/kg/day for 8 weeks (Figure 1A). To evaluate their running performance, we performed the exhaustive treadmill test and measured the time and distance they ran until exhaustion. Notably, mice supplemented with APIC, irrespective of dosage used (100 mg/kg/day and 200 mg/kg/day), exhibited significantly prolonged running times and covered greater distances compared to the vehicle control mice (Figure 1B,C). These findings indicate that APIC has a positive impact on improving running endurance.

To further investigate whether the enhancement in running endurance is associated with alterations in energy substrate utilization, we measured the respiratory exchange ratio (RER). Interestingly, the supplementation of APIC resulted in a decreased RER compared to the control mice (Figure 1D). This suggests that the mixture of soy isoflavones and L-carnitine promotes lipid utilization as an energy source during running. VO_2_ had no significant difference between groups (Figure 1E). Notably, these improvements in running endurance and whole-body energy metabolism occurred without significant changes in food intake, body weight, or lean body mass (Figure 1F–H).

### 3.2. APIC Supplementation Promotes the Transition from Glycolytic to Oxidative Myofibers

**Figure 2:**
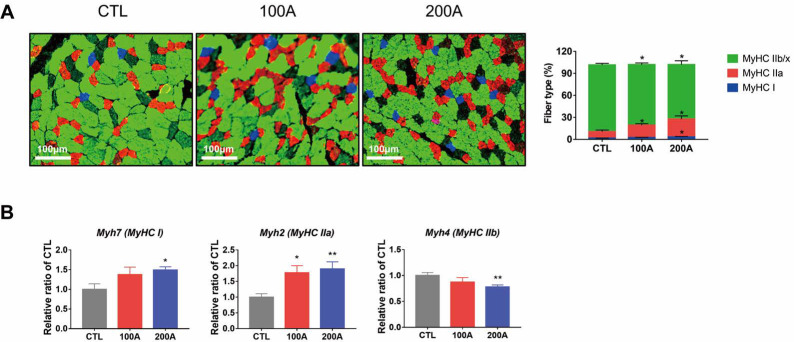
Effects of APIC supplementation on muscle fiber-type composition in the gastrocnemius muscles. (**A**) Cross-sections of gastrocnemius muscle were immunostained for fiber-typing (MyHC I (blue), IIa (red), IIb (green), and IIx (unstained)). The proportion of muscle fibers of different types was quantified (*n* = 6). (**B**) The expression of fiber-type genes was analyzed by qPCR. (*n* = 6–8). Values are mean ± S.E.M., * *p* < 0.05, and ** *p* < 0.01. CTL, vehicle; 100 A, APIC 100 mg/kg/day; 200 A, APIC 200 mg/kg/day.

Improved endurance is strongly linked to a higher percentage of oxidative myofibers, specifically type I and type IIa fibers [3]. Therefore, we assessed the composition of each fiber type in the gastrocnemius muscles. APIC supplementation led to an increase in the abundance of type I and IIa myofibers and a decrease in the abundance of type IIx and IIb fibers (Figure 2A). Similarly, mRNA levels of type I and IIa myofiber genes, namely *Myh7* and *Myh2*, respectively, were elevated in the APIC group compared to the control mice. Conversely, mRNA levels of type IIb myofiber gene, *Myh4,* were lower in the APIC group compared to the control mice (Figure 2B). In addition, we analyzed the distribution of the muscle fiber cross-sectional area (CSA). The APIC groups exhibited a higher frequency of smaller fibers and a decrease in average CSA (Figure S1), suggesting a shift from type IIx and IIb myofibers toward type I and IIa fibers [30,31].

### 3.3. Effect of APIC Supplementation on Biochemical Parameters

**Figure 3:**
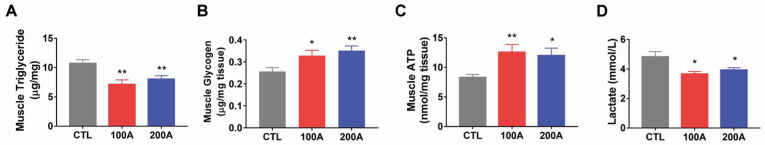
Effect of APIC supplementation on biochemical parameters in gastrocnemius muscles and blood. (**A**) Intramuscular triglyceride, (**B**) intramuscular glycogen, and (**C**) ATP levels in gastrocnemius muscles, as well as (**D**) blood lactate levels, were measured (*n* = 6–8). Values are mean ± SEM. * *p* < 0.05 and ** *p* < 0.01. CTL, vehicle; 100 A, APIC 100 mg/kg/day; 200 A, APIC 200 mg/kg/day.

The APIC group exhibited lower intramuscular triglyceride levels compared to the control mice (Figure 3A), suggesting a potential increase in lipid utilization in skeletal muscle. APIC administration also increased muscle glycogen content (Figure 3B). Additionally, intramuscular ATP levels were elevated with APIC administration (Figure 3C), while blood lactate levels were decreased (Figure 3D).

### 3.4. APIC Supplementation Activates AMPK and Upregulates Downstream Target Proteins

To investigate the molecular mechanisms underlying the enhancement of endurance capacity by APIC supplementation, we examined the gene and protein expression of AMPK and its downstream target proteins.

**Figure 4:**
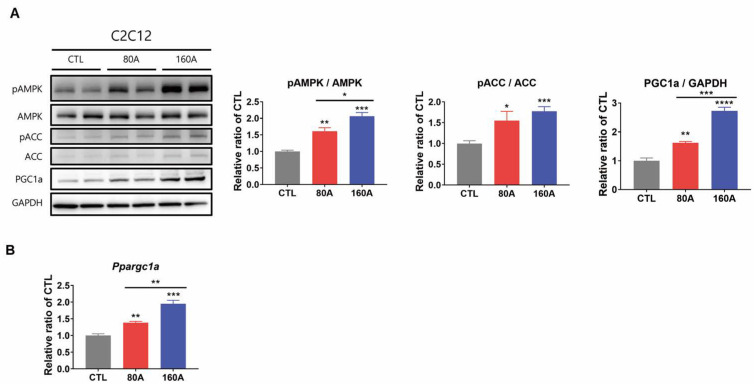
Effect of APIC supplementation on AMPK phosphorylation and downstream target proteins in vitro. (**A**) Immunoblot analysis of proteins involved in AMPK signaling in C2C12 myotubes (*n* = 6). (**B**) Relative mRNA expression levels of *Ppargc1a* (*n* = 6). Both proteins and RNAs were extracted from C2C12 myotubes treated with either vehicle (CTL) or APIC (80 A, 80 µg/mL; 160 A, 160 µg/mL) for 24 h. Values are mean ± SEM. * *p* < 0.05, ** *p* < 0.01, *** *p* < 0.001, and **** *p* < 0.0001.

APIC treatment dose-dependently increased AMPK phosphorylation in C2C12 myotubes (Figure 4A). The phosphorylation of ACC, a direct target of AMPK, was also enhanced by APIC treatment at both concentrations (Figure 4A). This indicates the increased activation of AMPK. In addition, ACC phosphorylation by AMPK inhibits its enzyme activity, which can partially account for the increased lipid utilization in the skeletal muscle of APIC-supplemented mice. Activation of AMPK is known to induce the expression of peroxisome proliferator-activated receptor gamma coactivator 1-alpha (PGC-1α), a coactivator involved in muscle fiber transition and mitochondrial biogenesis. Consistently, APIC supplementation upregulated both the gene and the protein expression of PGC-1α (Figure 4A,B). Individual treatments of isoflavone-containing soybean embryo extract were also found to increase AMPK phosphorylation and downstream target protein levels (Figure S2A). Notably, the combined treatment with L-carnitine further increased phospho-AMPK and PGC-1 α levels.

**Figure 5:**
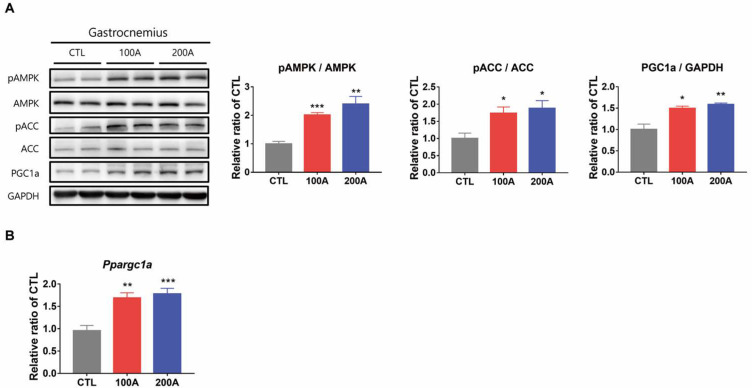
Effect of APIC supplementation on AMPK phosphorylation and downstream target proteins in vivo. (**A**) Immunoblot analysis of proteins involved in AMPK signaling in gastrocnemius muscles (*n* = 6–8). (**B**) Relative mRNA expression levels of *Ppargc1a* (*n* = 6–8). Both proteins and RNAs were extracted from gastrocnemius muscles of mice treated with vehicle (CTL) or APIC (100 A, 100 mg/kg/day; 200 A, 200 mg/kg/day) for eight weeks and subjected to acute exercise for 30 min before sacrifice. Values are mean ± SEM. * *p* < 0.05, ** *p* < 0.01, and *** *p* < 0.001.

These findings were further confirmed in vivo. Mice in both APIC groups exhibited elevated protein levels of phospho-AMPK, phospho-ACC, and PGC-1α (Figure 5A), as well as increased mRNA levels of PGC-1α (Figure 5B).

### 3.5. APIC Supplementation Increases Mitochondrial Content and Oxidative Capacity

**Figure 6:**
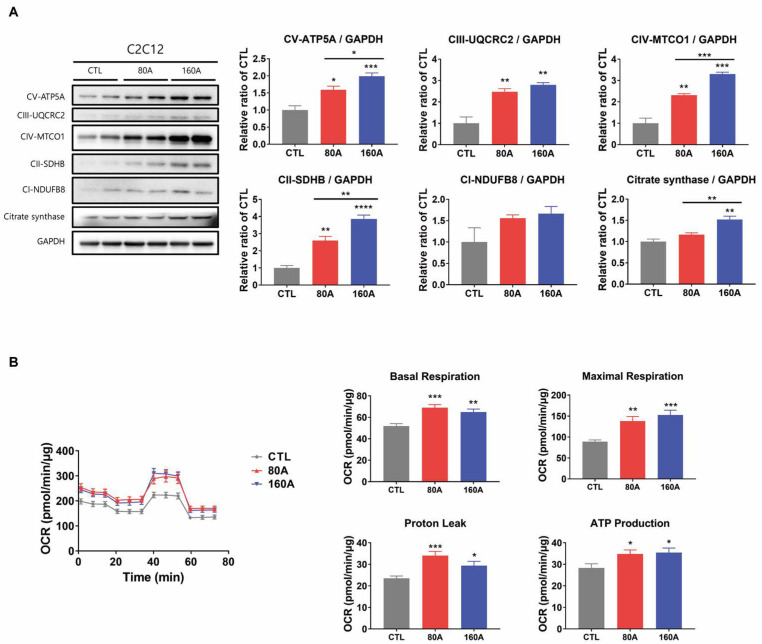
Effect of APIC supplementation on mitochondrial content and oxidative capacity in vitro. (**A**) Immunoblot analysis of proteins involved in mitochondrial oxidative phosphorylation and citrate synthase in C2C12 myotubes (*n* = 6). (**B**) Oxygen consumption rate of C2C12 myotubes (*n* = 8–10). Values are mean ± SEM. * *p* < 0.05, ** *p* < 0.01, *** *p* < 0.001, and **** *p* < 0.0001. CTL, vehicle; 80 A, APIC 80 µg/mL; 160 A, APIC 160 µg/mL.

We further investigated the effect of APIC on mitochondrial content, as higher mitochondrial content is a characteristic feature of oxidative fibers. In C2C12 myotubes, the protein expression levels of total OXPHOS and citrate synthase increased in a dose-dependent manner with APIC supplementation (Figure 6A). The expression of total OXPHOS increased with a single treatment of soybean embryo extract or L-carnitine, and the expression was higher in the combined treatment (Figure S2B).

**Figure 7:**
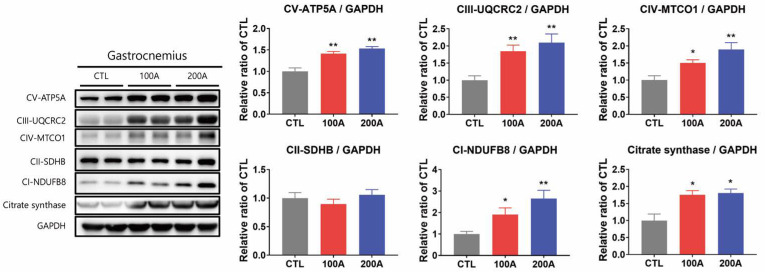
Effect of APIC supplementation on mitochondrial content and oxidative capacity in vivo. Immunoblot analysis of proteins involved in mitochondrial oxidative phosphorylation and citrate synthase in gastrocnemius muscles (*n* = 6–8). Values are mean ± SEM. * *p* < 0.05 and ** *p* < 0.01. CTL, vehicle; 100 A, APIC 100 mg/kg/day; 200 A, APIC 200 mg/kg/day.

Consistently, these protein levels were also elevated in the gastrocnemius muscles of APIC-supplemented mice compared to the control mice (Figure 7). Furthermore, in line with the increase in markers of mitochondrial content, APIC treatment resulted in an overall increase in oxygen consumption rates, including basal respiration, maximal respiration, proton leak, and ATP production in C2C12 myotubes (Figure 6B).

### 3.6. APIC Supplementation Increases the Expression of Genes and Proteins Involved in Fatty Acid Metabolism

**Figure 8:**
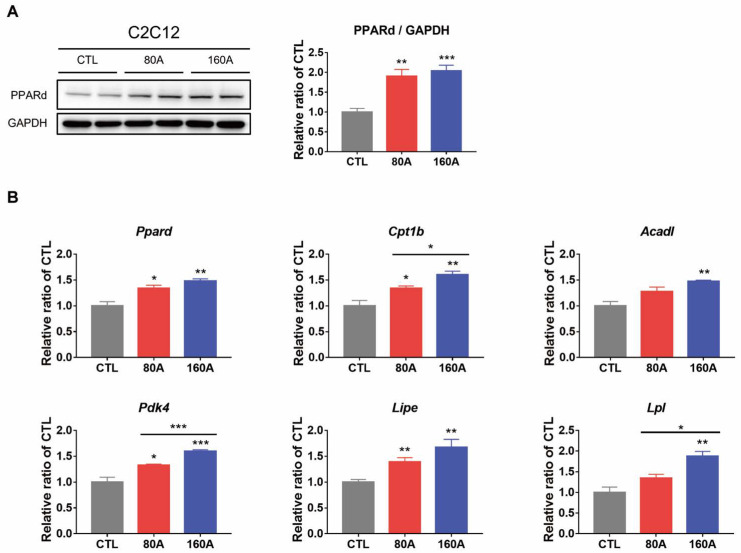
Effect of APIC supplementation on the expression of proteins and genes involved in fatty acid metabolism in vitro. (**A**) Protein expression level of PPARδ in C2C12 myotubes (*n* = 6). (**B**) Expression levels of genes involved in fatty acid metabolism in C2C12 myotubes (*n* = 6). Values are mean ± SEM. * *p* < 0.05, ** *p* < 0.01, and *** *p* < 0.001. CTL, vehicle; 80 A, APIC 80 µg/mL; 160 A, APIC 160 µg/mL.

In C2C12 myotubes, the protein expression level of peroxisome proliferator-activated receptor delta (PPARδ), a key regulator of fatty acid metabolism in skeletal muscle, was upregulated by APIC, along with its gene expression level (Figure 8A,B). APIC also significantly increased the expression of fatty acid oxidation-related genes, such as *Cpt1b*, *Acadl*, *Pdk4*, *Lipe*, and *Lpl* (Figure 8B).

Individual treatments of soybean embryo extract or L-carnitine were found to promote the induction of PPARδ and genes involved in fatty acid metabolism (Figure S2C). Notably, the combination APIC treatment exhibited synergistic effects, further enhancing the observed responses in cellular signaling and metabolic pathways (Figure S2).

**Figure 9:**
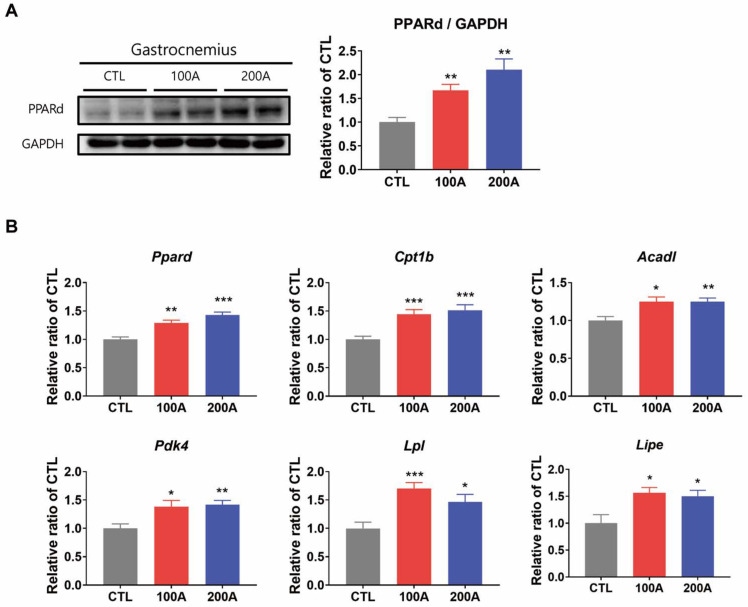
Effect of APIC supplementation on the expression of proteins and genes involved in fatty acid metabolism in vivo. (**A**) Protein expression level of PPARδ in gastrocnemius muscles (*n* = 6–8). (**B**) Relative expression levels of genes involved in fatty acid metabolism in gastrocnemius muscles (*n* = 6–8). Values are mean ± SEM. * *p* < 0.05, ** *p* < 0.01, and *** *p* < 0.001. CTL, vehicle p.o.; 100 A, APIC 100 mg/kg/day p.o.; 200 A, APIC 200 mg/kg/day p.o.

Similarly, APIC supplementation in vivo resulted in the upregulation of the same genes involved in fatty acid metabolism, accompanied by elevated protein and mRNA levels of PPARδ in gastrocnemius muscles (Figure 9A,B).

## 4. Discussion

This study demonstrates that APIC, a combination of soy isoflavone and L-carnitine, improves running endurance in mice. Mice supplemented with APIC exhibited increased running time and running distance to exhaustion, compared to the control group.

This improvement in running endurance in the APIC group was accompanied by muscle fiber-type transition. APIC supplementation increased the composition of oxidative fibers (type I and IIa) and reduced the composition of glycolytic fibers (type IIx and IIb) in gastrocnemius muscles. APIC supplementation also increased mitochondrial content and oxidative capacity both in vitro and in vivo. This further supports the increase in the composition of oxidative fibers in the APIC group, as oxidative fibers exhibit high levels of mitochondria and high oxidative metabolism [5]. We also observed that supplementation of APIC lowered RER in mice. A high RER value, close to 1.0, indicates a predominant utilization of carbohydrates as the primary source of energy, whereas a low RER value, around 0.7, suggests fat as the primary fuel source [32]. In line with the observed low RER value in the APIC-treated groups, there was a decrease in skeletal muscle triglyceride levels after exercise, indicating increased lipid utilization as fuel for energy [33]. Moreover, the upregulation of genes associated with fatty acid oxidative metabolism in skeletal muscles with APIC administration provides further evidence for APIC-induced metabolic reprogramming. Additionally, lower concentrations of blood lactate, a metabolite that can be generated from the conversion of pyruvates through glycolytic stimulation, in the APIC group suggest an increase in oxidative mitochondrial metabolism. It is worth noting that blood lactate accumulation has also been reported to negatively correlate with fat oxidation [34]. Comprehensively, these data suggest that APIC supplementation increases the abundance of oxidative fibers and their oxidative capacity, along with a shift in substrate utilization from carbohydrates toward fat.

Isoflavones have been reported to activate AMPK by phosphorylation [22,23,35]. AMPK promotes mitochondrial biogenesis through PGC-1α, and its gene expression is induced by AMPK activation [36]. In this study, we observed that APIC increased AMPK phosphorylation and protein expression of PGC-1α in gastrocnemius muscles. PGC-1α is a transcription coactivator that stimulates mitochondrial biogenesis and the conversion of glycolytic fibers to oxidative fibers [37]. Indeed, skeletal muscle-dependent PGC-1α overexpression increased the mRNA expression of mitochondrial enzymes and type I fiber markers [38]. This is consistent with an increase in mitochondrial content and the proportion of oxidative fibers in the skeletal muscle of the APIC group. AMPK also directly phosphorylates ACC, resulting in the reduction of malonyl-CoA to attenuate CPT1 inhibition [39]. This aligns with our observation of APIC-induced ACC phosphorylation. Based on our findings, we conclude that APIC activates AMPK, leading to an enhancement in mitochondrial biogenesis and fatty acid metabolism. The observed activation of AMPK signaling pathways by APIC suggested that APIC has the potential to be developed as an exercise mimetic.

L-carnitine is a major substance in APIC, along with soy isoflavones. It is notable that, in a recent study, L-carnitine supplementation in exercise-trained mice enhanced running endurance, but did not change the phosphorylation level of AMPK [12]. Instead, L-carnitine upregulated muscle fatty acid oxidative metabolism, including the gene expression of CPT1 and PPARδ in exercise-trained mice [12]. PPARδ is a key regulator of fatty acid metabolism [40,41]. A previous study reported that 8 weeks of PPARδ agonist treatment enhanced running endurance by shifting muscle energy substrate usage from glucose to fatty acid [9]. PPARδ also has been reported to upregulate genes involved in fatty acid metabolism, such as CPT1 and PDK4, in skeletal muscle [9,16]. In this study, APIC also upregulated the expression of PPARδ, CPT1, and PDK4. Accordingly, APIC-induced improvement in running endurance may be due to increased expression of PPARδ and the subsequent promotion of fatty acid oxidative metabolism. Interestingly, PPARδ agonist did not alter muscle fiber-type composition [9]; however, muscle-specific overexpression of PPARδ increased type I fiber and mitochondrial biogenesis [42]. This indicates the complex mechanism of PPARδ in governing fiber-type switching. While previous research has demonstrated isoflavones’ ability to bind to PPAR alpha and PPAR gamma [43], it is essential to explore their potential interaction with PPARδ to gain a comprehensive understanding of their molecular mechanisms and effects on cellular pathways.

The present study suggests that the effect of APIC on improving running endurance is mediated through the activation of AMPK and the increase in PPARδ expression. However, further studies are required to investigate the in vivo synergistic effect of soy isoflavone and L-carnitine on running endurance. The in vitro individual treatments with soy embryo extract (API) and L-carnitine led to an increase in the levels of pAMPK, pACC, mitochondrial proteins, and PPARδ. Remarkably, when the two compounds were combined, the effects were found to be additive, indicating a potential synergistic action, suggesting a potential cooperative interaction in enhancing cellular energy regulation and mitochondrial function. In this regard, previous studies have reported a synergistic anti-obesity effect of soy isoflavone and L-carnitine. In mice fed a high-fat diet, supplementation with soy isoflavone (or genistein) and L-carnitine for 12 weeks synergistically reduced body weight gain and body fat percentages, along with a decrease in serum lipid concentrations [25,44]. Furthermore, in the same study, the gene expression level of CPT1 in the liver was synergistically increased by the supplementation of genistein with L-carnitine [44]. The authors suggested that the synergistic anti-obesity effect of soy isoflavone and L-carnitine may be attributed to the promotion of fatty acid oxidation via CPT1. Accordingly, soy isoflavone and L-carnitine may have a synergistic effect on running endurance by boosting fatty acid oxidation. 

The current study found upregulation of genes associated with fatty acid oxidative metabolism and highlights the potential impact of APIC on muscle lipid metabolism. However, it is important to note that our investigation did not include data on muscle lipid concentration beyond triglyceride levels. Further research using metabolic tracing and lipidomics studies will be essential to comprehensively understand the effects of APIC on lipid metabolism in muscle tissue and its contribution to the observed improvements in running endurance. Additionally, exploring the effects of APIC on muscle strength could provide crucial insights into the mechanisms underlying the observed enhancement in running endurance. Future studies are needed to address this aspect to better understand the physiological effects of APIC and its potential impact on athletic performance.

In addition to the AMPK and PPAR delta-mediated effects of APIC, it is plausible that the observed improvements in muscle endurance may be mediated, at least in part, through the estrogen receptor signaling pathways [45]. Bioactive isoflavones, including phytoestrogens, have the ability to exert their effects through estrogen receptors [46], and it is known that estrogen receptor signaling plays a role in regulating skeletal muscle homeostasis and exercise capacity. Therefore, investigating the effects of APIC on estrogen-receptor-mediated signaling could be informative in understanding its involvement in the improvement of running endurance.

## 5. Conclusions

In summary, our study demonstrated that APIC supplementation leads to improved running endurance in mice through APMK and PPARδ signaling pathways. We observed that APIC enhances mitochondrial biogenesis, facilitates a shift from glycolytic to oxidative fiber types, and improves fatty acid metabolism. Collectively, these findings highlight the potential of APIC as a promising intervention for enhancing exercise performance and suggest its potential application in promoting metabolic adaptations associated with endurance exercise training. Further investigation into the precise molecular mechanisms underlying the observed effects of APIC will contribute to a better understanding of its potential as an exercise mimetic and may pave the way for the development of novel therapeutic strategies for enhancing endurance and metabolic fitness.

## Figures and Tables

**Table 1:** Primer sequences used for qPCR.

Gene	Forward 5′-3′	Reverse 5′-3′
*Ppia*	GTGGTCTTTGGGAAGGTGAA	TTACAGGACATTGCGAGCAG
*Ppargc1a*	ACACCTGTGACGCTTTCGCTG	AAGGACACGCTGTCCCATGA
*Ppard*	GGCAGCCTCAACATGGAATG	TCGAGCTTCATGCGGATTGT
*Cpt1b*	ATGCTCCGAGGCATTTGTCA	GGTCAGCTGGCCATGGTATT
*Acadl*	GGCGATTTCTGCCTGTGAGTTC	GCTGTCCACAAAAGCTCTGGTG
*Pdk4*	GTCGAGCATCAAGAAAACCGTCC	GCGGTCAGTAATCCTCAGAGGA
*Lipe*	GCTGGGCTGTCAAGCACTGT	GTAACTGGGTAGGCTGCCAT
*Lpl*	GCGTAGCAGGAAGTCTGACCAA	AGCGTCATCAGGAGAAAGGCGA

## Data Availability

The data provided in this research are available within the article.

## Supplementary Materials

The following supporting information can be downloaded at: https://www.mdpi.com/article/10.3390/nu15173678/s1, Figure S1. Analysis of myofiber cross-sectional area (CSA) in gastrocnemius muscles. Values are mean ± SEM. **** *p* < 0.0001. Figure S2. Synergistic effects of soybean embryo extract and L-carnitine co-treatments on AMPK signaling, mitochondrial content, and fatty acid metabolism in vitro. C2C12 myotubes were treated with either vehicle (CTL), soybean embryo extract (API, 91.4 μg/mL), L-carnitine (CAR, 68.6 μg/mL) or APIC (160 μg/mL) for 24 h. The treated amount of API and L-carnitine was determined based on their contents in APIC (in the ratio of 4:3) (A) Immunoblot analysis of proteins involved in AMPK signaling and (B) mitochondrial oxidative phosphorylation and (C) PPARδ in C2C12 myotubes (*n* = 6). (D) Expression levels of genes involved in fatty acid metabolism in C2C12 myotubes (*n* = 6). Values are mean ± SEM. * *p* < 0.05, ** *p* < 0.01, *** *p* < 0.001, and **** *p* < 0.0001. CTL, vehicle; API, soybean embryo extract; CAR, L-carnitine; APIC, APIC 160 μg/mL.
