# Supplementary material for: A Combination of Soy Isoflavone and L-Carnitine Improves Running Endurance in Mice
Source: Nutrients. 2023 Aug 22;15(17):3678. doi: 10.3390/nu15173678 (PMC10489700; doi:10.3390/nu15173678)
Supplement: Supplementary file 1 [file nutrients-15-03678-s001.zip › nutrients-2523358-supplementary.pdf]

Supplementary Figures

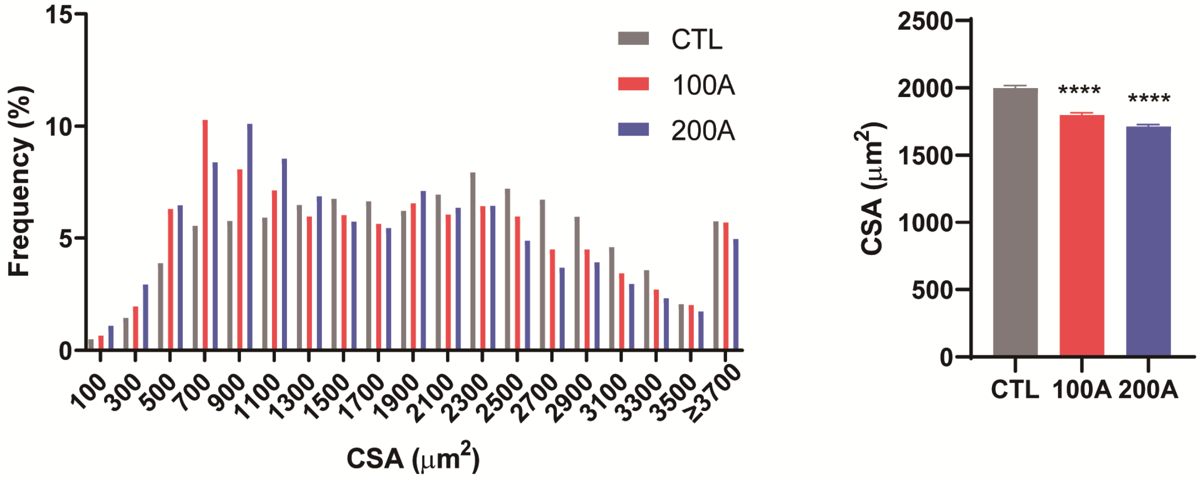

Figure S1. Analysis of myofiber cross-sectional area (CSA) in gastrocnemius muscles. Values are mean ± SEM. **** $p < 0.0001$.

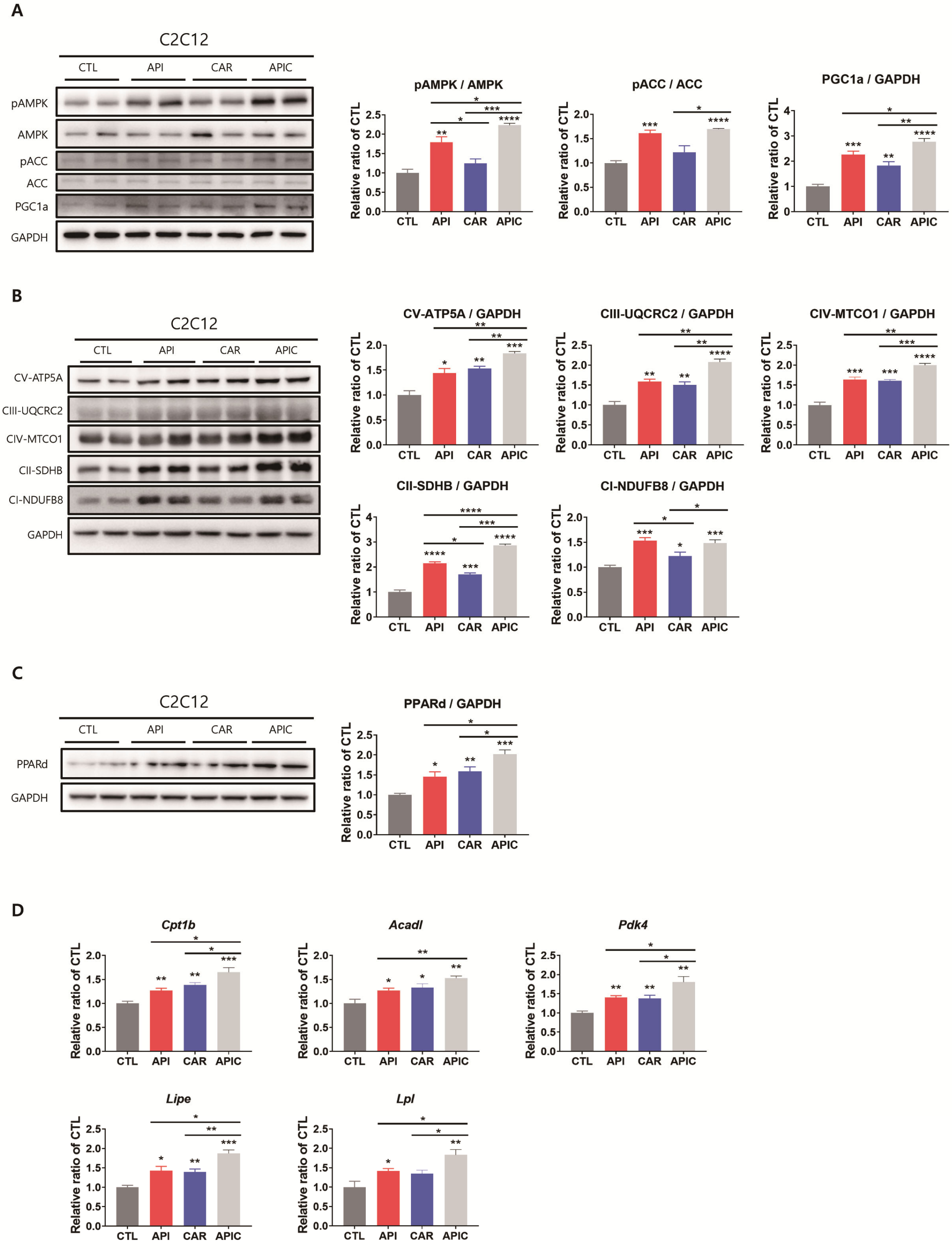

Figure S2. Synergistic effects of soybean embryo extract and L-carnitine co-treatments on AMPK signaling, mitochondrial content, and fatty acid metabolism in vitro. C2C12 myotubes were treated with either vehicle (CTL),

soybean embryo extract (API, 91.4 μg/mL), L-carnitine (CAR, 68.6 μg/mL) or APIC (160 μg/mL) for 24 h. The treated amount of API and L-carnitine was determined based on their contents in APIC (in the ratio of 4:3) (**A**) Immunoblot analysis of proteins involved in AMPK signaling and (**B**) mitochondrial oxidative phosphorylation and (**C**) PPARδ in C2C12 myotubes (n = 6). (**D**) Expression levels of genes involved in fatty acid metabolism in C2C12 myotubes (n = 6). Values are mean ± SEM. * $p < 0.05$, ** $p < 0.01$, *** $p < 0.001$, and **** $p < 0.0001$. CTL, vehicle; API, soybean embryo extract; CAR, L-carnitine; APIC, APIC 160 μg/mL.
